# Expression profiling of genes regulated by TGF-beta: Differential regulation in normal and tumour cells

**DOI:** 10.1186/1471-2164-8-98

**Published:** 2007-04-11

**Authors:** Prathibha Ranganathan, Animesh Agrawal, Raghu Bhushan, Aravinda K Chavalmane, Ravi Kiran Reddy Kalathur, Takashi Takahashi, Paturu Kondaiah

**Affiliations:** 1 Department of Molecular Reproduction, Development and Genetics, Indian Institute of Science, Bangalore 560012, India; 2 Division of Molecular Carcinogenesis, Centre for Neurological Diseases and Cancer, Nagoya University Graduate School of Medicine, Japan

## Abstract

**Background:**

TGF-beta is one of the key cytokines implicated in various disease processes including cancer. TGF-beta inhibits growth and promotes apoptosis in normal epithelial cells and in contrast, acts as a pro-tumour cytokine by promoting tumour angiogenesis, immune-escape and metastasis. It is not clear if various actions of TGF-beta on normal and tumour cells are due to differential gene regulations. Hence we studied the regulation of gene expression by TGF-beta in normal and cancer cells.

**Results:**

Using human 19 K cDNA microarrays, we show that 1757 genes are exclusively regulated by TGF-beta in A549 cells in contrast to 733 genes exclusively regulated in HPL1D cells. In addition, 267 genes are commonly regulated in both the cell-lines. Semi-quantitative and real-time qRT-PCR analysis of some genes agrees with the microarray data. In order to identify the signalling pathways that influence TGF-beta mediated gene regulation, we used specific inhibitors of p38 MAP kinase, ERK kinase, JNK kinase and integrin signalling pathways. The data suggest that regulation of majority of the selected genes is dependent on at least one of these pathways and this dependence is cell-type specific. Interestingly, an integrin pathway inhibitor, RGD peptide, significantly affected TGF-beta regulation of Thrombospondin 1 in A549 cells.

**Conclusion:**

These data suggest major differences with respect to TGF-beta mediated gene regulation in normal and transformed cells and significant role of non-canonical TGF-beta pathways in the regulation of many genes by TGF-beta.

## Background

TGF-β is a multifunctional cytokine that plays important patho-physiological roles in mammals. There are three mammalian isoforms that are involved in several developmental processes as has been shown by the knock-out mice models [1]. TGF-β has a major role to play in the initiation and progression of cancer. This is supported by several studies which have shown defects in various components of the TGF-β signalling pathway in many cancers [2]. TGF-β has a dual role in carcinogenesis [3]. Initially it acts as a tumour suppressor and causes growth arrest of epithelial cells and cells in the early stages of cancer [4]. But in an established tumour, TGF-β exerts an effect which is favourable for the survival, progression and metastasis of the tumour [5,6] by promoting epithelial-mesenchymal transition (EMT), angiogenesis and escape from immune surveillance [7]. Studies using mouse models have shown that an intact TGF-β signalling is essential for the metastasis of breast cancer [8,9]. These observations indicate that the normal epithelial cells show differential response to TGF-β as compared to the tumour they give rise to. Supporting this, it has been shown that prostate tumour cells show invasion in response to TGF-β and not non-tumourigenic cells [10]. Differential gene expression mediated by TGF-β has been reported in tumour cells and normal cells. For example, in response to TGF-β, tumour cells show increase in the production of proteases and down regulation of the inhibitors of proteases, whereas this is not observed in the normal cells [11-14]. However, there is no clear understanding of the mechanism (s) responsible for differential responses of various cell types to TGF-β. Since a role for TGF-β has been established in several pathological conditions, this pathway is a very attractive target for therapeutic intervention. This requires identification of targets of TGF-β in different cell-types and their mechanism of regulation, particularly in un-transformed and transformed cells. In this study, we show differential regulation of several genes by TGF-β in two different cell-lines, HPL1D and A549 and also propose a significant role for the MAP kinase pathway in TGF-β mediated gene regulations.

## Results

### Gene expression profiling of HPL1D and A549 cells in response to TGF-β

**Figure 1:**
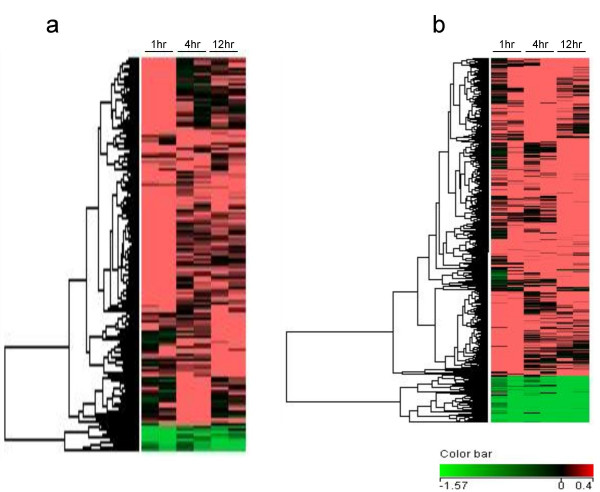
**Hierarchical clustering of genes regulated by TGF-β in (a) HPL1D and (b) A549 cells**. The genes regulated by TGF-β (described in materials and methods) were clustered using Avadis 3.3 Prophetic software. The data was clustered on rows by one way complete linkage – hierarchical clustering using Euclidean distance metric. Microarrays have been done in duplicate on RNAs isolated from cells after 1, 4 and 12 hours of treatment with TGF-β with paired controls at every time point. The dendrogram on the left shows the different clusters into which the TGF-β regulated genes segregated. Red indicates genes induced by TGF-β treatment, green indicates genes repressed by TGF-β treatment and black suggests no regulation.

To identify the TGF-β regulated genes in normal and tumour cells, we chose HPL1D and A549 cells. HPL1D is an immortalized lung epithelial cell-line that is growth inhibited by TGF-β, similar to many epithelial cells [15]. A549 is a lung adenocarcinoma cell-line that has been known to respond to TGF-β treatment [16]. The cells were treated with human recombinant TGF-β 1 for 1, 4 and 12 hours and the RNAs extracted from these cells were used for microarray experiments using human 19 k arrays. Genes which were either up (> 1.3 fold) or down regulated (< 0.33 fold) at any one of the time points have been considered as regulated by TGF-β in the respective cell-line. In HPL1D, 1000 genes were regulated by TGF-β treatment and of these, 917 genes were up regulated and 83 genes were down regulated. In A549, 2024 genes were regulated by TGF-β and of these, 1714 genes were up regulated and 310 genes were down regulated by TGF-β treatment. The log_2 _transformed data of the genes regulated by TGF-β in HPL1D and A549 cells are shown as hierarchical cluster diagrams (Fig. 1a &1b). The expression profiling data of HPL1D and A549 in response to TGF-β has been submitted to Gene Expression Omnibus (accession number- GSE7436).

### Comparison of gene expression profiles of HPL1D and A549 in response to TGF-β

**Figure 2:**
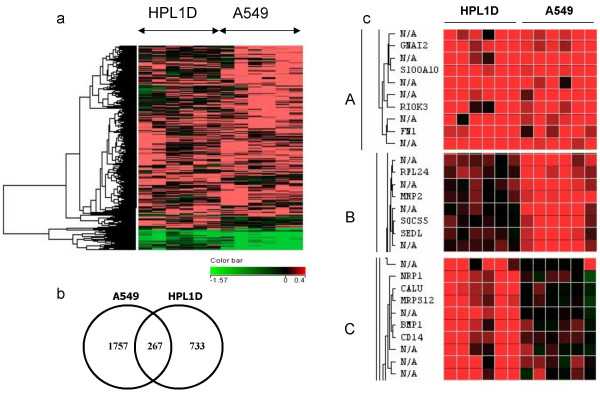
**Comparison of genes regulated by TGF-β in HPL1D and A549 cells**. **a, ***Hierarchical clustering of genes regulated by TGF-β in HPL1D and A549 cells *Genes regulated by TGF-β in HPL1D and A549 cells were clustered using Avadis 3.3 software. For this comparison only those genes for which expression values are available in both the cell-lines have been considered. The dendrogram on the left shows different clusters of genes segregated according to the pattern of regulation in the two cell-lines. **b, ***Venn diagram showing the genes regulated by TGF-β either common or exclusively to the cell-lines *and **c**, *Expanded clusters showing sub-classes of genes regulated by TGF-β in HPL1D and A549 ***(**derived from Figure 2a); **A, **Genes regulated by TGF-β in both HPL1D and A549 (267 genes); **B**, Genes regulated by TGF-β in A549 and not in HPL1D (1757 genes); **C, **Genes regulated by TGF-β in HPL1D and not in A549 (733 genes).

When the gene expression profiles of HPL1D and A549 cell-lines in response to TGF-β 1 were compared, it was found that 267 genes were regulated in both the cell-lines in a similar manner but with different kinetics. The list of genes regulated commonly in both HPL1D and A549 is shown in additional file 1 (Table S1). A hierarchical cluster diagram is shown in figure 2a and a Venn diagram depicting the number of genes commonly and differentially regulated in A549 and HPL1D are shown in figure 2b. 1757 genes are exclusively regulated by TGF-β in A549 cells, (see additional file 2 Table S2) and 733 genes are exclusively regulated by TGF-β in HPL1D cells (see additional file 3, Table S3). Representative clusters of each of the sub-classes of genes are shown in figure 2c.

### Validation of microarray results by qRT-PCR

**Figure 3:**
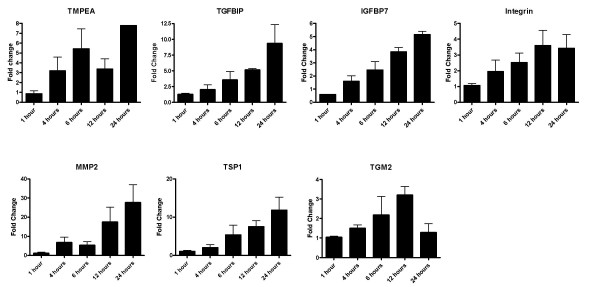
**qRT-PCR analyses of selected genes (TMEPA, TGFBIP, IGFBP7, Integrin αV, TSP-1, MMP2, TGM2) with respect to TGF-β treatment in A549 cells**. The cells were grown to 90% confluence, washed with serum free medium and treated with 5ng/ml TGF-β 1 for 1, 4, 6, 12 and 24 hours along with untreated controls at each time-point. Two microgram of total RNA from each treatment was reverse transcribed and cDNA equivalent to 10ng total RNA was used for the PCR reactions. The graphs represent the fold change over untreated controls after normalization with the expression of RPL35a. The bars show mean ± SD of two experiments of the PCR reactions done in duplicates.

**Figure 4:**
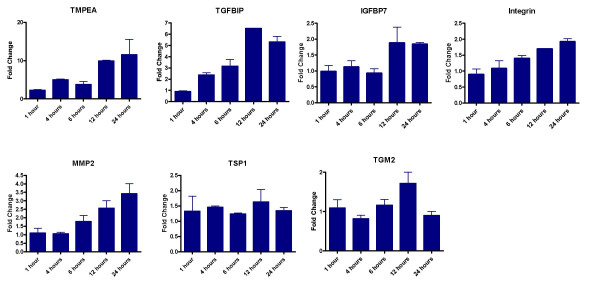
**qRT-PCR analyses of selected genes (TMEPA, TGFBIP, IGFBP7, Integrin αV, TSP-1, MMP2, TGM2) with respect to TGF-β treatment in HPL1D cells**. The cells were grown to 90% confluence, washed with serum free medium and treated with 5ng/ml TGF-β 1 for 1, 4, 6, 12 and 24 hours along with untreated controls at each time-point. Two microgram of total RNA from each treatment was reverse transcribed and cDNA equivalent to 10ng total RNA was used for the PCR reactions. The graphs represent the fold change over untreated controls after normalization with the expression of RPL35a. The bars show mean ± SD of two experiments of the PCR reactions done in duplicates.

The microarray data have been validated by real time and semi-quantitative RT-PCR. TMEPA, TGFBIP, IGFBP7, Integrin α V, TSP-1, MMP2 and TGM2 were studied by real time qRT-PCR as described in the methods. The primer sequences used for qRT-PCR are shown in table 1. Any gene is considered as regulated by TGF-β if qRT-PCR results showed > two fold regulation as compared to untreated cells. As shown in figure 3 and 4, TMEPA, TGFBIP and MMP2 are regulated by TGF-β 1 in both HPL1D and A549 cell-lines. In HPL1D, induction of TGFBIP and TMEPA by TGF-β is seen after 4 hours of treatment and increases with prolonged incubation. The induction of MMP2 by TGF-β is seen 6 hours onwards, whereas, there is no regulation of the genes IGFBP7, TSP-1, TGM2 after TGF-β treatment. Integrin αV, showed a 1.8 fold regulation after 24 hours of treatment with TGF-β. In A549, the induction of all these genes except TGM2 is observed after 4 hours of treatment and increase progressively with time, whereas, the induction of TGM2 increases till 12 hours and then declines. In addition, regulation of few other genes by TGF-β such as fibronectin 1, ZF36, S100A2, Tβ RI, ACTA2, T-plastin, keratin 7 and v-jun were assessed by semi-quantitative RT-PCR (See additional file 4, Fig. S1A). The data suggest that all these genes are regulated by 1.5 – 2.0 fold in HPL1D cells (See additional file 4, Fig. S1B). However, in A549 cells, the regulation of ZF36, ACTA2, Keratin 7, Tβ RI and v-jun is > 2.0 fold whereas, FN1, T-plastin and S100A2 is 1.5–2.0 fold (See additional file 4, Fig. S1C).

### Functional categories of genes regulated by TGF-β in HPL1D and A549

The lists of the regulated genes were fed into DAVID database (database for annotation, visualization and integrated discovery [17]. Based on the functions of the genes assigned by DAVID, genes were classified into the following categories. In HPL1D, TGF-β regulated genes belong to (a) regulation of actin cytoskeleton (table 2), (b) Focal adhesion (table 3) and (c) Wnt signalling pathways (table 4). In A549, TGF-β modulated genes belong to (a) MAP kinase signalling (table 5), (b) tight junction (table 6), (c) adherans junction (table 7) (d) focal adhesion (table 8) (e) insulin signalling (table 9) (f) regulation of actin cytoskeleton (table 10) and (g) Wnt signalling (table 11).

### Effect of inhibiting the MAPK pathways on the TGF-β mediated gene regulation

**Figure 5:**
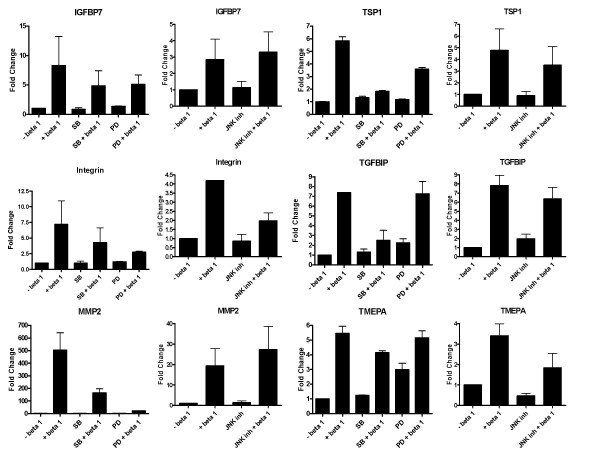
**Effect of inhibition of the MAP kinase pathways on the regulation of gene expression by TGF-β in A549 cells**. The cells were grown to 90% confluence, washed with serum free medium, treated with 10 μM p38 inhibitor (SB203580), ERK inhibitor (PD98059) or JNK inhibitor 1 hour prior to treatment with TGF-β. The cells were harvested after 18 hours following TGF-β treatment and processed for qRT-PCR as described in Figure 3.

**Figure 6:**
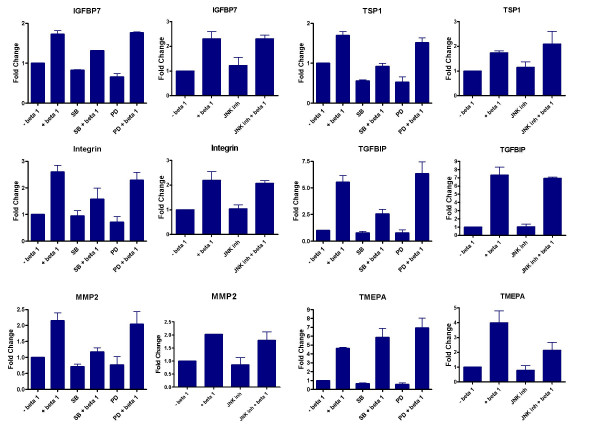
**Effect of inhibition of the MAP kinase pathways on the regulation of gene expression by TGF-β in HPL1D cells**. The cells were grown to 90% confluence, washed with serum free medium, treated with 10 μM p38 inhibitor (SB203580), ERK inhibitor (PD98059) or JNK inhibitor 1 hour prior to treatment with TGF-β. The cells were harvested after 18 hours following TGF-β treatment and processed for qRT-PCR as described in Figure 3.

TGF-β modulates gene expression through phosphorylation of SMAD proteins. However, several studies show a role for MAP kinase pathway in TGF-β mediated gene regulation both in a SMAD dependent and independent manner [18-20]. Hence, inhibitors of MAP kinase pathway components, SB203580 (p38), PD98059 (ERK) and JNK inhibitor 1-L form were used to assess the role of p38, ERK and JNK pathways in the regulation of gene expression by TGF-β. In A549 cells, regulation of IGFBP7 by TGF-β is independent of the p38, ERK and the JNK pathways. Induction of Integrin αV, MMP2, TMEPA and TGM2 by TGF-β is partially dependent on the ERK pathway; and MMP2, TGFBIP, TGM2 and TSP-1 regulation by TGF-β is partially dependent on the p38 pathway. The induction of Integrin αV is partially affected by blocking the JNK pathway (Fig. 5). In HPL1D cells, Intgerin αV, MMP2 and TGFBIP regulation by TGF-β is affected by blocking the p38 MAP kinase pathway, none of the gene regulations by TGF-β seem to be affected by blocking the ERK pathway and the induction of TMEPA by TGF-β is dependent on the JNK pathway (Fig. 6).

### Effect of blocking the Integrin-linked signalling pathway in A549 cells

**Figure 7:**
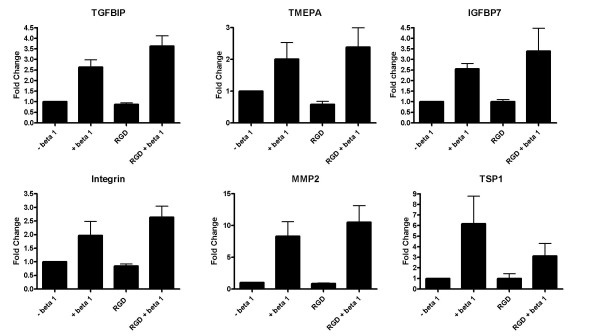
**Effect of blocking the Integrin-linked signalling on the regulation of gene expression by TGF-β in A549 cells**. The cells were grown to 90% confluence, washed with serum free medium, treated with 500 μg/ml GRGDNP peptide 3 hours prior to treatment with TGF-β. The cells were harvested after 12 hours following TGF-β treatment and processed for qRT-PCR as described in Figure 3.

**Figure 8:**
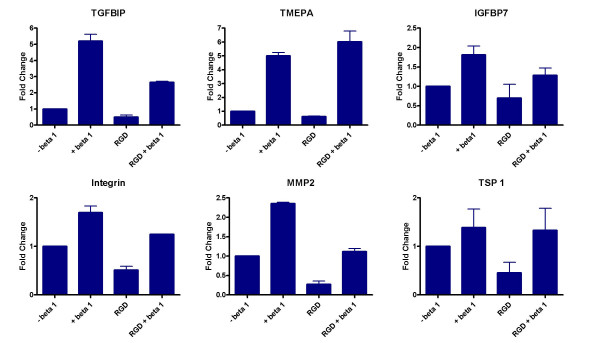
**Effect of blocking the Integrin-linked signalling on the regulation of gene expression by TGF-β in HPL1D cells**. The cells were grown to 90% confluence, washed with serum free medium, treated with 500 μg/ml GRGDNP peptide 3 hours prior to treatment with TGF-β. The cells were harvested after 12 hours following TGF-β treatment and processed for qRT-PCR as described in Figure 3.

Apart from the various signalling pathways and phenomena modulated by TGF-β in both normal and tumour cells, a pathway which is likely be differentially regulated is the Integrin-αV linked signalling pathway. Integrin αV is induced by TGF-β in A549 cells to about 3.5 fold as compared to 1.8 fold in HPL1D. Integrin αV is known to mediate some actions of TGF-β [21,22]. In order to test if some of the genes regulated by TGF-β are due to activation of Integrin αV, the activation of this pathway was blocked by treating the cells with 500 μg/ml of GRGDNP peptide, a known Integrin pathway inhibitor, prior to treatment with TGF-β. It was found that the induction of TSP-1, which is a TGF-β regulated gene only in A549 cells, is affected by blocking the integrin-linked signalling pathway (Figure 7 and Figure 8). Blocking this pathway had no effect on the regulation of the other genes tested. This suggests that the Integrin pathway is also responsible for differential regulation of some genes by TGF-β in the two cell-types, HPL1D and A549.

## Discussion

Most actions of TGF-β are brought about by regulation of gene expression. The genes, which are regulated and the way they are regulated are largely dependent on the cell-type under consideration. Over the past few years, there have been several independent transcriptome analyses of cells in response to TGF-β treatment. In cells of epithelial origin like human keratinocytes and mouse breast epithelial cells, TGF-β modulates genes involved in EMT [23,24]. In lung fibroblasts and A549 cells, TGF-β treatment results in the induction of extracellular matrix genes which are responsible for fibrosis as in the case of idiopathic pulmonary and alveolar epithelial fibrosis [25,26]. Studies using various cells such as cervical cancer cells, corneal epithelial cells, rat intestinal epithelial cells, and dermal fibroblasts show that regulation of gene expression by TGF-β is cell-type specific [27-30]. Considering the dual role of TGF-β on normal and transformed cells, wherein it confers growth inhibition and apoptosis to normal epithelial cells but aids growth and metastasis of tumour cells [4-7], it is essential to identify the genes and/or biochemical pathways regulated by TGF-β in normal and transformed cells. An understanding of these differences may allow identification of therapeutic targets for diseases involving TGF-β signalling pathway.

With this aim, expression profiling of genes in response to TGF-β was performed in a lung adenocarcinoma cell-line (A549) and a matched immortalized lung epithelial cell-line (HPL1D). The experimental design includes treatment of the respective cells with 5 ng/ml TGF-β in serum free conditions for 1, 4 and 12 hrs and extract RNA for microarray experiments. In our experiments, we have used two arrays for each RNA (time point) and hence essentially these are technical duplicates. As a consequence although duplicate arrays were performed for each time point, effectively the sample size equals to 1 at each time point since they are technical duplicates. Due to this, it is possible that many changes that are seen could be due to variations in the experimental protocols, rather than real biological differences. To overcome this, we have performed qRT-PCR validation of several genes on biological replicates that agrees with the microarray data for the respective genes. Our data showed similar regulation of 267 genes in HPL1D and A549 cells by TGF-β. This suggests that the genes commonly regulated in both HPL1D and A549 are not tumour specific. Some of these genes were also reported to be regulated by TGF-β in other studies using microarray in various cell-types [23,24,27] suggesting that these TGF-β regulated genes are not tumour cell specific but generally regulated by TGF-β. While some 1757 genes are exclusively regulated by TGF-β in A549, only 733 genes are exclusively regulated in HPL1D cells. The reasons for this differential response are not known. However, some of the genes exclusively regulated in A549 such as Integrin αV, thrombospondin 1, α2macroglobulin have been shown to aid tumour survival, maintenance and metastasis [31-33]. In contrast, in HPL1D, TGF-β regulates tumour suppressor genes like WT1, ECM proteins like collagen which are responsible for arrest of cell growth and apoptosis. This differential gene regulation in normal and tumour cells may explain the dual role of TGF-β in carcinogenesis. When the genes regulated by TGF-β in these two cell-lines were categorized based on their annotated functions, it was found that signalling pathways like MAP kinase, focal adhesion, Wnt signalling are regulated by TGF-β in both the cell-lines. On the other hand, Integrin αV was found to be differentially regulated in A549 and HPL1D cells.

TGF-β actions on cells are to a large extent are carried out by the phosphorylation of SMAD 2/3 by activated TGF-β type I receptor upon TGF-β signalling. Several genes that are transcriptionally regulated by TGF-β contain a SMAD complex binding element (SBE). However, many genes are reported to be regulated by TGF-β through an AP1 element [34,35]. Using SMAD2/SMAD3 knock out fibroblast cell-lines, SMAD 2 and 3 independent actions of TGF-β have been reported earlier [36]. In addition, SMAD independent signalling by TGF-β mediated by other pathways like the MAP kinases namely the p38, ERK and the JNK pathways is also reported [18-20,37-40]. Activation of the p38 and the JNK pathways by TGF-β involves a TGF-β activated kinase called TAK1 (reviewed in [41,42]). However, the ERK pathway activation by TGF-β is not well characterized. Also, cross talk between SMAD mediated signalling and PKC pathway has been reported [43]. Another pathway that has been implicated in TGF-β actions is the integrin signalling pathway. It has been shown that activation of integrin β 1 along with αV is important for the EMT mediated by TGF-β [22]. Although, Integrin αV is known to mediate some actions of TGF-β in some cell-types [21,22,44-46], the genes regulated by TGF-β through this pathway have not been characterized. Our data suggest substantial induction of integrin αV by TGF-β in only A549 cells and marginally in HPL1D cells. This could be an important difference between these two cell-lines for the regulation of gene expression mediated by TGF-β via this pathway. In order to understand the mechanism of regulation of some of the previously validated genes identified in the microarray experiments, specific inhibitors for the three branches of the MAP kinase pathway namely the p38, the ERK and the JNK pathways were used prior to TGF-β treatment of the cells and expression of these genes was studied by real time qRT-PCR estimation. The results suggested that in A549 cells, active MAP kinase pathways are involved in the regulation of more number of genes with relatively higher magnitude by TGF-β as compared to HPL1D cells (Fig. 5&6). There could be several reasons for this observation: 1] It is known that the MAP kinases are constitutively active in highly malignant cells due to autocrine activation of MAP kinase pathways [47], PI3K/Akt pathways [48], constitutive activation or amplification of receptors such as EGFR, PDGFR etc., mechanisms [49]. MAP kinase pathway involvement in the TGF-β mediated gene regulation is well established and the constitutive activation of MAP kinase pathway in transformed cells could be one of the reasons for more number of genes regulated by TGF-β in A549 cells. 2] It could be due to activation of Integrin pathway in transformed cells. It has been reported that Integrin mediated FAK/ILK activation is required for the regulation of EMT [22,50]. Our data suggesting regulation of Thrombospondin 1 which is an EMT marker [51] by TGF-β via integrin pathway, could be an example of this pathway's importance in TGF-β mediated gene regulation at least with respect to genes that mediate EMT. Taken together, our data highlights the importance of MAP kinase and integrin pathways in the regulation of majority of genes by TGF-β at least in transformed cells. The role of SMAD pathway in the regulation of these genes that are affected by the MAP kinase and integrin αV inhibitors is not known. It is possible that some of these regulations that depend on non-SMAD pathways may also need activated SMAD pathway. One difference between normal and tumour cells could be the absence of an intact SMAD signalling pathway in tumour cells. However, in A549 cells, no defects in the SMAD pathway have been reported. In addition, we confirmed the presence of intact SMAD pathway in A549 cells by SMAD dependent regulation of promoter activity by TGF-β (See additional file 5, Fig. S2). Based on our data, we propose that activated MAP kinase pathway could be one of the essential determining factors for the various differential actions of TGF-β in tumour cells.

A major question that remains to be addressed is whether the TGF-β mediated differential gene expression patterns in HPL1D and A549 cells is due to the distinct phenotypic characteristics of the respective cell-lines. From the previous studies, it is well established that TGF-β influences the growth and apoptosis of untransformed epithelial cells such as lung epithelial cells [52]. At least the growth inhibition by TGF-β has been less sensitive in the presence of serum [53] suggesting that activation of signalling pathways by serum may antagonize TGF-β mediated growth inhibition. Most apoptosis studies using TGF-β have also been carried out in minimal serum conditions (0.2–0.5 %). It remains to be established if apoptotic cell death mediated by TGF-β could be reversed in the presence of serum although it can be speculated that reversal of growth inhibition may allow survival of cells. One of the most important actions of the serum is activation of mitogenic pathways mediated by growth factors. Also, it has been shown that serum stimulation leads to activation of MAP kinase pathways [54]. Our data demonstrates that the effect of TGF-β is more pronounced in A549 cells as compared to HPL1D cells and one of the reasons for this could be due to constitutively active MAP kinase pathway in tumour cells. This is evident from our data that shows loss of TGF-β regulation of many genes following MAP kinase pathway specific inhibitors in A549 cells. A549 cells harbour a mutation at the 12^th ^codon of K-ras proto-oncogene [55]. This mutation renders constitutive activation of RAS protein resulting in active MAP kinase pathway in these cells. In HPL1D cells, there is no report of any mutation in the RAS genes or constitutively active MAP kinase pathway. It is possible that this may be one of the major differences responsible for the differential regulation of genes by TGF-β in these cell-lines. HPL1D cells are growth inhibited by TGF-β [15]. The differential regulation of certain tumour suppressor genes (WT1, S100A2, TSSC1), cell cycle related genes (CDC34, CHEK1) and extracellular matrix genes (fibronectin, collagen IV and VIII) by TGF-β in HPL1D cells (see additional file 3, Table S3) points to a growth regulatory activity of TGF-β in these cells. On the other hand, as discussed previously, genes that are known to play pro-tumourigenic roles such as thrombospondin, integrins, transglutaminase, α2-macroglobulin etc. are regulated in A549 cells (see additional file 2, Table S2). Taken together, the differential regulation of genes in A549 and HPL1D cells could be due to pro-tumourigenic or growth inhibitory roles of TGF-β on these cells.

## Conclusion

In conclusion, by microarray and real time RT-PCR experiments, we show that 1] TGF-β regulates more number of genes in transformed cells as compared to non-transformed cells; 2] evidence for differential regulation of gene expression in normal and tumour cells by TGF-β; and 3] that involvement of MAP kinase pathways may be one of the major mechanisms of TGF-β actions.

## Methods

### Cell-lines and cultures

A549, a lung adenocarcinoma cell-line was cultured in DMEM (Sigma-Aldrich, USA) with 10% foetal bovine serum (FBS), 100 units/ml penicillin and 100 μg/ml streptomycin, 2.5 μg/ml fungizone (Invitrogen Life Sciences, USA). HPL1D, an immortalized lung epithelial cell-line was cultured in Ham's F-12 supplemented with 5% FBS, 5 μg/ml bovine insulin, 5 μg/ml human transferrin, 10^-7^M hydrocortisone, 2 × 10^-10^M tri-iodo thyronine and 20 ng/ml EGF [15]. All the cell-lines were maintained at 37°C in a humid atmosphere with 5% CO_2_.

### Treatments

The cell-lines were grown to 90% confluence in the respective growth media followed by serum free washes (3 times) for 24 hours and then treated with 5 ng/ml TGF-β1 (R&D systems, USA) for different intervals of time. For signal transduction pathway inhibitor experiments, cells were pre-treated with 10 μM SB203580 (p38 inhibitor), 10 μM PD98050 (ERK inhibitor), and 10 μM JNK inhibitor I (L-form) (all from Calbiochem) for 1 hour, and 500 μg/ml GRGDNP peptide (custom synthesized) for 2–3 hours prior to TGF-β treatments.

### RNA isolation, cDNA labelling and microarray analysis

Total RNA was isolated from cells using TRI reagent (Sigma-Aldrich, USA) according to manufacturer's protocol. The RNA was re-precipitated with 0.3 M sodium acetate (pH 5.2) and ethanol, further purified using RNAeasy columns (Qiagen, GmbH, Germany). The RNA quantity and quality were assessed by OD_260 _and OD_280 _measurements on a spectrophotometer and the integrity was determined by MOPS-formaldehyde gel. The labelling of the cDNA for microarray was done using Micromax Direct Labelling kit (Perkin Elmer Life Sciences Inc. USA) according to manufacturer's instructions. For each labelling reaction, 20 μg of total RNA was used. The control RNA was labelled with Cy3-dUTP and the TGF-β 1 treated RNA with Cy5-dUTP. Both the labelled products were mixed and precipitated with 0.3 M sodium acetate (pH 5.2) and ethanol. A small quantity of the precipitated labelled cDNA was assessed for labelling efficiency on a 1% agarose gel and scanned using the Typhoon 9210 scanner (GE Life Sciences). 160 μl of hybridisation buffer (Ultrahyb, Sigma-Aldrich, USA) was added to the probe, incubated at 75°C for 5 minutes and then added to the human 19 K array (University Health network, Toronto). The hybridisation was carried out in the GeneTAC Hyb Station (Genomic Solutions, UK) at 65° for 4 hours, 60° for 4 hours and 55° for 10 hours. The slides were then washed with medium stringency (2× SSC and 0.1% SDS), high stringency (0.1× SSC and 0.1% SDS) and post wash (0.1× SSC) buffers for 5 minutes each, dried and scanned using a scanner (Scanarray Express, Perkin Elmer Life Sciences, USA).

### Microarray image and data analyses

All the image analyses have been done using the Quantarray software (Perkin Elmer Life Sciences, USA). Filtering and compilation of data have been done using Microsoft Excel and Microsoft Access. Spots of compromised quality and with low intensity were eliminated from the analysis. The data was normalized by LOWESS method (Avadis 3.1, Strand Life Sciences, India), Cy5:Cy3 ratios were established and log_2 _values were calculated. Hybridizations of RNA from each time point were performed on duplicate (technical duplicate) arrays and only those genes, which showed consistent regulation, have been considered for analysis. Genes which show log_2_ratios greater than 0.37 (1.3 fold induced) or less than -1.5 (3 fold down regulated) in the duplicate arrays at any one of the time points have been considered as regulated by TGF-β. The cut-off values for regulated genes were decided based on our observation of the data where genes that were known to be up regulated by TGF-β were showing > 1.3 and < 0.33 fold difference in the microarray.

### Real Time PCR analyses

For cDNA synthesis, two micrograms of total RNA was reverse transcribed using the ABI cDNA Archive kit (Applied Biosystems, USA). Complementary DNA (cDNA) equivalent to 10 ng of total RNA was used for all the PCR reactions. The sequences of the primers are shown in Table 1. All the PCR reactions have been done using Dynamo SYBR green mix (Finnzymes, Finland) in ABI Prism 7900HT sequence detection system (Applied Biosystems, USA). The analysis has been done using SDS 2.1 software (Applied Biosystems, USA). For normalization of RT-PCR data, RPL35a expression (which was previously found to be unchanged in microarray experiments) was used and fold over control at each of the time point has been calculated as follows

δCT = CT_gene_-CT_RPL_

δδCT = δCT_treated_-δCT_untreated_

Fold Change = 2^-δδCT^.

## Authors' contributions

PR designed and executed the experiments, interpreted the data and prepared the manuscript; AA, RB and RKRK analysed the microarray data and prepared the gene lists and figures; AC performed the real time PCR experiments and analysis of data; TT provided the support in the design of experiments and PK conceived the study, participated in its design and coordination and helped to draft the manuscript. All authors read and approved the final manuscript.

## Supplementary Material

Additional file 1 List of genes commonly regulated by TGF-β in HPL1D and A549 cell-lines. A list of genes, which are regulated by TGF-β in both HPL1D and A549 cell-lines along with the fold changes at 1, 4 and 12 hrs of treatment. Click here for file

Additional file 2 List of genes regulated by TGF-β in only A549 cell-line. A list of genes, which are regulated by TGF-β in A549 alone along with the fold changes at 1, 4 and 12 hrs of treatment. Click here for file

Additional file 3 List of genes regulated by TGF-β only in HPL1D cell-line. A list of genes, which are regulated by TGF-β in HPL1D cell-line alone along with the fold changes at 1, 4 and 12 hrs of treatment. Click here for file

Additional file 4 Semi-quantitative RT-PCR analyses of selected genes with respect to regulation by TGF-β in A549 and HPL1D cells. The respective cell-lines were grown to 90% confluence (as described in methods section), washed with serum free medium, treated with 5 ng/ml TGF-β 1 for 1, 4, 6, 12 and 24 hours. Each treatment also has untreated cells as control at each time-point. Two microgram of total RNA from each treatment was reverse transcribed and cDNA equivalent to 20 ng total RNA was used for the PCR reactions. All PCR reactions were done under non saturating conditions. The products were resolved on 2% agarose gel and the gel pictures were taken on Kodak Image station 440CF. The band intensities were quantified using Kodak 1D 3.6 software. A, ethidium bromide staining pattern of the PCR products. B and C graphs representing the fold change over untreated controls after normalization with the expression of RPL35a, in HPL1D and A549 cells respectively. Click here for file

Additional file 5 Induction of pSBE-Luc activity by TGF-β in A549 cells. For SBE-luc induction by TGF-β in A549 cells, twenty five thousand cells were plated in 24 well dishes 16–24 hours prior to transfection. 200 ng of pSBE-luc plasmid and 1.25 ng of pRL-CMV construct (Renilla luciferase, for transfection normalization) were transfected using Effectene reagent (Qiagen GmbH, Germany) in serum free conditions for 12 hrs. The cells were recovered in medium containing 10% FBS for 24 hours, washed with serum free medium for 24 hours (3 changes) and then treated with 5 ng/ml TGF-β for 18 hours. The cells were then lysed and lysates were used for dual-luciferase assay (Promega inc, USA). The ratio of the firefly-luciferase to renilla-luciferase has been plotted on the y-axis. Click here for file

## Figures and Tables

**Table 1:** List of genes selected for validation by real-time RT-PCR

**Gene Name**	**Fold regulation in microarray**		**Primer sequences**
	**HPL1D cells**	**A549 cells**	

TGF-β induced protein 68 kDa (TGFBIP)	1.8	2.8	F 5' tgtgtgctgaagccatcgttg 3' R 5' ccggcttgtctgaaaaggtca 3'
Trans membrane prostate androgen induced RNA (TMEPA)	1.5	3.08	F5'ttcattccctgtcctcattgg3' R5'gcacaacagccatggaatca3'
Insulin like growth factor binding protein 7 (IGFBP7)	1.49	2.72	5' ggtccttccatagtgacgcc 3' 5' tctgaatggccaggttgtcc 3'
Matrix metalloprotease 2 (MMP2)	1.0	1.32	5' ctgatggcacccatttacacc 3' 5' gcctcgtataccgcatcaatc 3'
Transglutaminase 2 (TGM2)	1.2	3.27	5' ccatgaccagaacagcaacct3' 5' tgacctccgcaaagacaaag 3'
Thrombospondin 1 (TSP-1)	1.1	2.24	5' ccggcgtgaagtgtactagcta3' 5' tgcacttggcgttcttgtt 3'
Integrin αV	1.1	2.73	5' caggcttgcaacccattct 3' 5' cctggcgagtttggttttct 3'
RPL35a			5' gggtacagcatcactcgga 3' 5' acgcccgagatgaaacag 3'

The genes were selected based on any or both of the following criteria. 1. Genes that showed differential regulation between cell-lines and show maximum regulation in at least one of the cell-lines, 2. Genes that showed regulation in the array and are known as TGF-β regulated genes in other studies. The primer sequences are shown 5' to 3' and F and R denote forward and reverse primers respectively.

**Table 2:** TGF-β regulated genes (17) in HPL1D involved in regulation of actin cytoskeleton

**Acc No**	**DAVID Gene Name**	**1 hr**	**4 hr**	**12 hr**
W20454	FIBRONECTIN 1	1.23 ± 0	1.36 ± 0.04	2.1 ± 0.19
H58824	DIAPHANOUS HOMOLOG 1 (DROSOPHILA)	0.84 ± 0.1	1.57 ± 0.19	1 ± 0.11
N46724	RHO GUANINE NUCLEOTIDE EXCHANGE FACTOR (GEF) 7	1.4 ± 0.07	0.96 ± 0.21	1 ± 0.07
AA045207	ACTIN RELATED PROTEIN 2/3 COMPLEX, SUBUNIT 5, 16KDA	1.3 ± 0.09	1.3 ± 0.23	1.55 ± 0.12
BM924824	RAS-RELATED C3 BOTULINUM TOXIN SUBSTRATE 1 (RHO FAMILY, SMALL GTP BINDING PROTEIN RAC1)	1.11 ± 0.08	1.14 ± 0.03	1.55 ± 0.07
BQ017489	ACTININ, ALPHA 1	1.42 ± 0.03	1.02 ± 0.11	1.53 ± 0.2
H73128	INTEGRIN, BETA 5	1.06 ± 0.03	1.21 ± 0.03	1.47 ± 0.11
W56632	CD14 ANTIGEN	1.46 ± 0	1.15 ± 0	1.45 ± 0.06
AA046597	INTEGRIN, BETA 4	1.63 ± 0.14	1.57 ± 0	1.43 ± 0.07
H23109	FIBROBLAST GROWTH FACTOR 1 (ACIDIC)	1.55 ± 0.1	0.94 ± 0.08	1.42 ± 0.2
AA010526	GLUCOCORTICOID RECEPTOR DNA BINDING FACTOR 1	1.37 ± 0.26	1.29 ± 0.07	1.36 ± 0.02
W67710	PLATELET-DERIVED GROWTH FACTOR BETA POLYPEPTIDE (SIMIAN SARCOMA VIRAL (V-SIS) ONCOGENE HOMOLOG)	1.53 ± 0.07	1.17 ± 0.02	1.2 ± 0.01
AA037763	WAS PROTEIN FAMILY, MEMBER 2	1.41 ± 0.08	1.08 ± 0.02	1.18 ± 0.05
W39107	RAS HOMOLOG GENE FAMILY, MEMBER A	1.37 ± 0.05	1.07 ± 0.04	1.14 ± 0.16
AL598940	MYOSIN, LIGHT POLYPEPTIDE 1, ALKALI; SKELETAL, FAST	1.36 ± 0.05	1.06 ± 0.12	1.07 ± 0.02
AA131272	INTEGRIN, ALPHA 8	1.19 ± 0	1.38 ± 0.06	0.96 ± 0.02
H83405	FYVE, RHOGEF AND PH DOMAIN CONTAINING 1 (FACIOGENITAL DYSPLASIA)	0.45 ± 0.01	0.52 ± 0.17	0.25 ± 0

The list in the table shows the regulated genes as revealed by the DAVID tool. The columns show Genbank accession no., gene name, and fold change with respect to untreated controls at 1 hr, 4 hr and 12 hr time points following TGF-β treatment respectively. The regulation shown against each gene is depicted as fold change ± S.E. with respect to untreated cells. Each experimental value of the duplicates of the respective genes is shown in tables S1 and S3 (see additional file 1 and 3). The number in the parenthesis of the title represents the number of genes in the table.

**Table 3:** TGF-β regulated genes (19) in HPL1D involved in focal adhesion

**Acc No**	**DAVID Gene Name**	**1 hr**	**4 hr**	**12 hr**
AA031376	PLACENTAL GROWTH FACTOR, VASCULAR ENDOTHELIAL GROWTH FACTOR-RELATED PROTEIN	1.18 ± 0.47	1.36 ± 0.05	1.16 ± 0.04
AA046597	INTEGRIN, BETA 4	1.63 ± 0.14	1.57 ± 0	1.43 ± 0.07
AA131272	INTEGRIN, ALPHA 8	1.19 ± 0	1.38 ± 0.06	0.96 ± 0.02
AW959692	VASCULAR ENDOTHELIAL GROWTH FACTOR C	1.15 ± 0.17	1.34 ± 0.02	1.44 ± 0.31
N74737	COLLAGEN, TYPE IV, ALPHA 2	0.84 ± 0.01	1 ± 0.04	1.51 ± 0.01
AA030029	PROTEIN KINASE C, ALPHA	1.3 ± 0	1.14 ± 0.03	1.1 ± 0.07
AL079948	THROMBOSPONDIN 1	0.78 ±	1.82 ± 0.09	2.5 ± 0.1
H73128	INTEGRIN, BETA 5	1.06 ± 0.03	1.21 ± 0.03	1.47 ± 0.11
BM924824	RAS-RELATED C3 BOTULINUM TOXIN SUBSTRATE 1 (RHO FAMILY, SMALL GTP BINDING PROTEIN RAC1)	1.11 ± 0.08	1.14 ± 0.03	1.55 ± 0.07
BM801832	CAVEOLIN 1, CAVEOLAE PROTEIN, 22 KDA	1.09 ± 0.11	1.4 ± 0.01	1.47 ± 0
W67710	PLATELET-DERIVED GROWTH FACTOR BETA POLYPEPTIDE (SIMIAN SARCOMA VIRAL (V-SIS) ONCOGENE HOMOLOG)	1.53 ± 0.07	1.17 ± 0.02	1.2 ± 0.01
W20454	FIBRONECTIN 1	1.23 ± 0	1.36 ± 0.04	2.1 ± 0.19
AA029359	LAMININ, BETA 2 (LAMININ S)	1.54 ± 0.06	1.17 ± 0.08	1.02 ± 0.06
W39107	RAS HOMOLOG GENE FAMILY, MEMBER A	1.37 ± 0.05	1.07 ± 0.04	1.14 ± 0.16
AA043301	COLLAGEN, TYPE IV, ALPHA 1	1.4 ± 0.19	1.52 ±	1.52 ± 0.03
BQ017489	ACTININ, ALPHA 1	1.42 ± 0.03	1.02 ± 0.11	1.53 ± 0.2
AA010526	GLUCOCORTICOID RECEPTOR DNA BINDING FACTOR 1	1.37 ± 0.26	1.29 ± 0.07	1.36 ± 0.02
R14058	PROTEIN KINASE C, BETA 1	1.34 ± 0.02	0.81 ± 0.1	0.94 ± 0.03
H58824	DIAPHANOUS HOMOLOG 1 (DROSOPHILA)	0.84 ± 0.1	1.57 ± 0.19	1 ± 0.11

For description see legend of table 2

**Table 4:** TGF-β regulated genes (15) in HPL1D involved in wnt signaling

**Acc No**	**DAVID Gene Name**	**1 hr**	**4 hr**	**12 hr**
N92026	PROTEIN PHOSPHATASE 3 (FORMERLY 2B), CATALYTIC SUBUNIT, BETA ISOFORM (CALCINEURIN A BETA)	0.92 ± 0.06	1.31 ± 0.01	1.05 ± 0.11
H82025	PRICKLE-LIKE 1 (DROSOPHILA)	1.79 ± 0.18	1.17 ± 0.02	1.21 ±
AA044665	F-BOX AND WD-40 DOMAIN PROTEIN 11	1.24 ± 0.18	1.14 ±	1.42 ± 0.08
AA030029	PROTEIN KINASE C, ALPHA	1.3 ± 0	1.14 ± 0.03	1.1 ± 0.07
AA203216	PROTEIN PHOSPHATASE 2 (FORMERLY 2A), REGULATORY SUBUNIT B (PR 52), BETA ISOFORM	1.26 ± 0	1.07 ± 0.2	1.5 ± 0.04
H79065	PRICKLE-LIKE 2 (DROSOPHILA)	1.38 ± 0.07	1.04 ± 0.28	1.01 ± 0.14
BM921730	PROTEIN PHOSPHATASE 2 (FORMERLY 2A), REGULATORY SUBUNIT A (PR 65), BETA ISOFORM	1.48 ± 0.01	1.19 ± 0.06	1.17 ± 0.01
R23027	CASEIN KINASE 1, ALPHA 1	1.28 ± 0.08	1.46 ± 0.01	1.26 ± 0.08
BM924824	RAS-RELATED C3 BOTULINUM TOXIN SUBSTRATE 1 (RHO FAMILY, SMALL GTP BINDING PROTEIN RAC1)	1.11 ± 0.08	1.14 ± 0.03	1.55 ± 0.07
BM311217	WINGLESS-TYPE MMTV INTEGRATION SITE FAMILY, MEMBER 4	1.26 ± 0.09	1 ± 0.06	1.34 ± 0.02
W86518	C-TERMINAL BINDING PROTEIN 2	1.33 ± 0.01	1.35 ± 0.24	1.13 ± 0.07
H70045	CALCIUM/CALMODULIN-DEPENDENT PROTEIN KINASE (CAM KINASE) II GAMMA	1.48 ± 0.16	1.1 ± 0.01	1.16 ± 0.05
W39107	RAS HOMOLOG GENE FAMILY, MEMBER A	1.37 ± 0.05	1.07 ± 0.04	1.14 ± 0.16
BE618078	CALCIUM/CALMODULIN-DEPENDENT PROTEIN KINASE (CAM KINASE) II BETA	1.58 ± 0.02	0.86 ± 0.05	1.02 ± 0.1
R14058	PROTEIN KINASE C, BETA 1	1.34 ± 0.02	0.81 ± 0.1	0.94 ± 0.03

For description see legend of table 2

**Table 5:** TGF-β regulated genes (38) in A549 involved in MAP kinase signalling

**Acc No**	**DAVID Gene Name**	**1 hr**	**4 hr**	**12 hr**
H09832	ACTIVIN A RECEPTOR, TYPE IB	1.01 ± 0.17	1.66 ± 0.29	1.17 ± 0.06
N27681	HEAT SHOCK 70 KDA PROTEIN 1A	1.12 ± 0.01	1.46 ± 0.18	1.42 ± 0.08
H73255	MITOGEN-ACTIVATED PROTEIN KINASE KINASE KINASE 6	0.84 ± 0.31	1.04 ± 0.07	1.49 ± 0.11
N42722	GUANINE NUCLEOTIDE BINDING PROTEIN (G PROTEIN), GAMMA 12	1.44 ± 0.04	1.5 ± 0.09	1.28 ± 0.17
AA039598	RIBOSOMAL PROTEIN S6 KINASE, 90 KDA, POLYPEPTIDE 1	1.16 ± 0.13	1.21 ± 0.02	1.32 ± 0.02
AA030029	PROTEIN KINASE C, ALPHA	1.33 ± 0.23	1.56 ± 0.08	1.36 ± 0.03
H73472	CELL DIVISION CYCLE 42 (GTP BINDING PROTEIN, 25 KDA)	1.24 ± 0.27	1.33 ± 0.07	1.56 ± 0.01
H62639	HEAT SHOCK 70 KDA PROTEIN 8	1.29 ± 0.01	1.68 ± 0.18	1.37 ± 0
H23019	TRANSFORMING GROWTH FACTOR, BETA RECEPTOR I (ACTIVIN A RECEPTOR TYPE II-LIKE KINASE, 53 KDA)	1.09 ± 0.01	2.53 ± 0.07	2.11 ± 0.41
AA088648	FIBROBLAST GROWTH FACTOR RECEPTOR 1 (FMS-RELATED TYROSINE KINASE 2, PFEIFFER SYNDROME)	1.45 ± 0.24	1.97 ± 0.08	1.77 ± 0.09
AV747676	V-AKT MURINE THYMOMA VIRAL ONCOGENE HOMOLOG 3 (PROTEIN KINASE B, GAMMA)	1.06 ± 0.13	1.46 ± 0.05	1.18 ± 0.01
AI500475	RAS GUANYL RELEASING PROTEIN 1 (CALCIUM AND DAG-REGULATED)	1.31 ± 0.05	1.37 ± 0	1.41 ± 0.07
R86053	NUCLEAR FACTOR OF KAPPA LIGHT POLYPEPTIDE GENE ENHANCER IN B-CELLS 2 (P49/P100)	0.99 ± 0.12	0.9 ± 0.07	1.44 ± 0.09
BE502112	RIBOSOMAL PROTEIN S6 KINASE, 90 KDA, POLYPEPTIDE 3	0.37 ± 0.1	0.29 ± 0.05	0.41 ± 0.06
H23109	FIBROBLAST GROWTH FACTOR 1 (ACIDIC)	0.94 ±	1.36 ± 0.01	1.13 ± 0.05
AA127934	PROTEIN KINASE, X-LINKED	1.21 ± 0.07	1.38 ± 0.01	1.35 ± 0.07
H89206	PROTEIN KINASE, CAMP-DEPENDENT, CATALYTIC, BETA	1.18 ± 0.08	1.42 ± 0.04	1.25 ± 0.03
R14348	MITOGEN-ACTIVATED PROTEIN KINASE KINASE KINASE 5	1.45 ± 0.09	1.22 ± 0.17	1.03 ± 0.06
BM924824	RAS-RELATED C3 BOTULINUM TOXIN SUBSTRATE 1 (RHO FAMILY, SMALL GTP BINDING PROTEIN RAC1)	1.39 ± 0	1.41 ± 0.06	1.28 ± 0.18
H11708	MITOGEN-ACTIVATED PROTEIN KINASE KINASE 4	1.38 ± 0.02	1.54 ± 0.1	1.24 ± 0.17
R36401	FILAMIN B, BETA (ACTIN BINDING PROTEIN 278)	0.96 ± 0.02	1.17 ± 0.11	1.33 ± 0.01
BI758537	MADS BOX TRANSCRIPTION ENHANCER FACTOR 2, POLYPEPTIDE C (MYOCYTE ENHANCER FACTOR 2C)	1.77 ± 0.42	1.25 ±	1.36 ± 0.09
W56249	ACTIVATING TRANSCRIPTION FACTOR 2	1.25 ± 0.13	1.5 ± 0.02	1.72 ± 0.18
AA034981	INTERLEUKIN 1 RECEPTOR, TYPE I	0.37 ± 0.08	0.4 ± 0.02	0.29 ± 0.04
AA056664	V-AKT MURINE THYMOMA VIRAL ONCOGENE HOMOLOG 1	1.34 ± 0.15	1.44 ± 0.05	1.36 ± 0.07
H20677	MITOGEN-ACTIVATED PROTEIN KINASE KINASE KINASE 7 INTERACTING PROTEIN 1	1.13 ± 0.15	0.96 ± 0.1	1.6 ± 0.01
BM999610	V-JUN SARCOMA VIRUS 17 ONCOGENE HOMOLOG (AVIAN)	1.57 ± 0.26	2.13 ± 0.01	1.66 ± 0.18
BE781203	CALCIUM CHANNEL, VOLTAGE-DEPENDENT, ALPHA 1G SUBUNIT	1.13 ± 0.03	1.02 ± 0	1.44 ± 0
T83272	RAS P21 PROTEIN ACTIVATOR 2	0.96 ± 0.08	1.12 ± 0.18	1.33 ± 0.02
T75436	MITOGEN-ACTIVATED PROTEIN KINASE 10	1.16 ± 0.17	1.22 ± 0.05	1.37 ± 0.07
W70006	CELL DIVISION CYCLE 25B	1.47 ± 0.1	1.15 ± 0.05	1.38 ± 0.08
R32409	PHOSPHOLIPASE A2, GROUP V	1.14 ± 0.27	1.27 ± 0.01	1.5 ± 0.07
AW961873	CALCIUM CHANNEL, VOLTAGE-DEPENDENT, GAMMA SUBUNIT 4	1.45 ± 0.15	1.23 ± 0.17	1.02 ± 0
BI752921	PLATELET-DERIVED GROWTH FACTOR RECEPTOR, BETA POLYPEPTIDE	1.43 ± 0.09	1.03 ± 0.02	1.09 ± 0.07
BQ055308	RIBOSOMAL PROTEIN L4	1.44 ± 0.05	1.26 ± 0.04	1.43 ± 0.08
R14058	PROTEIN KINASE C, BETA 1	1.54 ± 0.06	1.08 ± 0.01	1.15 ± 0
BQ016117	V-FOS FBJ MURINE OSTEOSARCOMA VIRAL ONCOGENE HOMOLOG	1.48 ± 0.26	1.44 ± 0.04	1.29 ± 0.06
W05234	CALCIUM CHANNEL, VOLTAGE-DEPENDENT, BETA 3 SUBUNIT	1.4 ± 0.07	1.15 ± 0.05	1.27 ± 0

The list in the table shows the regulated genes as revealed by the DAVID tool. The columns show Genbank accession no., gene name, and fold change with respect to untreated controls at 1 hr, 4 hr and 12 hr time points following TGF-β treatment respectively. The regulation shown against each gene is depicted as fold change ± S.E. with respect to untreated cells. Each experimental value of the duplicates of respective genes is shown in tables S1 and S2 (see additional files 1 and 2). The number in the parenthesis of the title represents the number of genes in the table.

**Table 6:** TGF-β regulated genes (21) in A549 involved in tight junction

**Acc No**	**DAVID Gene Name**	**1 hr**	**4 hr**	**12 hr**
AA027986	PROTEIN PHOSPHATASE 2 (FORMERLY 2A), CATALYTIC SUBUNIT, ALPHA ISOFORM	1.24 ± 0.15	1.65 ± 0.03	1.34 ± 0.11
H80401	V-YES-1 YAMAGUCHI SARCOMA VIRAL ONCOGENE HOMOLOG 1	0.3 ± 0.19	0.2 ± 0.07	0.29 ± 0.01
R52046	ACTININ, ALPHA 4	1.26 ± 0.68	1.21 ±	1.81 ± 0.21
AA030029	PROTEIN KINASE C, ALPHA	1.33 ± 0.23	1.56 ± 0.08	1.36 ± 0.03
BE738680	RAS HOMOLOG GENE FAMILY, MEMBER A	1.24 ± 0.11	1.4 ± 0.06	1.51 ± 0.03
H73472	CELL DIVISION CYCLE 42 (GTP BINDING PROTEIN, 25 KDA)	1.24 ± 0.27	1.33 ± 0.07	1.56 ± 0.01
N92643	CALCIUM/CALMODULIN-DEPENDENT SERINE PROTEIN KINASE (MAGUK FAMILY)	1.31 ±	1.45 ± 0.01	1.03 ± 0.01
BI828016	SPECTRIN, ALPHA, NON-ERYTHROCYTIC 1 (ALPHA-FODRIN)	1.33 ± 0.08	1.38 ± 0.01	1.93 ± 0.04
AV747676	V-AKT MURINE THYMOMA VIRAL ONCOGENE HOMOLOG 3 (PROTEIN KINASE B, GAMMA)	1.06 ± 0.13	1.46 ± 0.05	1.18 ± 0.01
AA031382	GUANINE NUCLEOTIDE BINDING PROTEIN (G PROTEIN), ALPHA INHIBITING ACTIVITY POLYPEPTIDE 2	1.23 ± 0.04	1.21 ± 0.05	1.65 ± 0.21
R14061	PAR-3 PARTITIONING DEFECTIVE 3 HOMOLOG (C. ELEGANS)	1.52 ± 0.07	1.12 ± 0.04	1.05 ± 0
AA056664	V-AKT MURINE THYMOMA VIRAL ONCOGENE HOMOLOG 1	1.34 ± 0.15	1.44 ± 0.05	1.36 ± 0.07
BI553762	CATENIN (CADHERIN-ASSOCIATED PROTEIN), ALPHA 2	1.36 ± 0.06	1.17 ± 0.03	1.88 ± 0.21
BM993684	CORTACTIN	1.63 ± 0.07	1.32 ± 0.11	1.26 ± 0.08
AA009869	ERYTHROCYTE MEMBRANE PROTEIN BAND 4.1 (ELLIPTOCYTOSIS 1, RH-LINKED)	1.19 ± 0.13	1.21 ± 0.05	1.39 ± 0.02
AA058385	TIGHT JUNCTION PROTEIN 2 (ZONA OCCLUDENS 2)	1.35 ± 0.15	1.36 ± 0.02	1.16 ± 0.18
AI375078	KIAA1634 PROTEIN	1.48 ± 0.02	1.25 ± 0.03	1.04 ± 0.01
BM978991	MEMBRANE ASSOCIATED GUANYLATE KINASE, WW AND PDZ DOMAIN CONTAINING 2	1.1 ± 0.05	1.15 ± 0	1.47 ± 0.05
N43838	ERYTHROCYTE MEMBRANE PROTEIN BAND 4.1-LIKE 3	1.4 ± 0.08	1.15 ± 0.14	1.15 ± 0.09
BQ017489	ACTININ, ALPHA 1	1.57 ± 0.22	1.24 ± 0.1	1.5 ± 0.1
R14058	PROTEIN KINASE C, BETA 1	1.54 ± 0.06	1.08 ± 0.01	1.15 ± 0

For description see legend of table 5

**Table 7:** TGF-β regulated genes (21) in A549 involved in adherans junction

**Acc No**	**DAVID Gene Name**	**1 hr**	**4 hr**	**12 hr**
H78804	SMAD, MOTHERS AGAINST DPP HOMOLOG 3 (DROSOPHILA)	1.22 ± 0.09	1.36 ± 0.02	1.15 ± 0.06
H09832	ACTIVIN A RECEPTOR, TYPE IB	1.01 ± 0.17	1.66 ± 0.29	1.17 ± 0.06
H80401	V-YES-1 YAMAGUCHI SARCOMA VIRAL ONCOGENE HOMOLOG 1	0.3 ± 0.19	0.2 ± 0.07	0.29 ± 0.01
R52046	ACTININ, ALPHA 4	1.26 ± 0.68	1.21 ±	1.81 ± 0.21
AA029516	TRANSCRIPTION FACTOR 7-LIKE 2 (T-CELL SPECIFIC, HMG-BOX)	1.25 ± 0.02	1.41 ± 0.02	1.14 ± 0.2
BE738680	RAS HOMOLOG GENE FAMILY, MEMBER A	1.24 ± 0.11	1.4 ± 0.06	1.51 ± 0.03
H73472	CELL DIVISION CYCLE 42 (GTP BINDING PROTEIN, 25 KDA)	1.24 ± 0.27	1.33 ± 0.07	1.56 ± 0.01
H23019	TRANSFORMING GROWTH FACTOR, BETA RECEPTOR I (ACTIVIN A RECEPTOR TYPE II-LIKE KINASE, 53 KDA)	1.09 ± 0.01	2.53 ± 0.07	2.11 ± 0.41
AA088648	FIBROBLAST GROWTH FACTOR RECEPTOR 1 (FMS-RELATED TYROSINE KINASE 2, PFEIFFER SYNDROME)	1.45 ± 0.24	1.97 ± 0.08	1.77 ± 0.09
R55134	TRANSCRIPTION FACTOR 7-LIKE 1 (T-CELL SPECIFIC, HMG-BOX)	1.45 ± 0.03	1.2 ± 0.1	1.27 ± 0.05
BM314539	CATENIN (CADHERIN-ASSOCIATED PROTEIN), DELTA 1	1.35 ± 0.04	1.21 ± 0.08	1.05 ± 0.06
R14061	PAR-3 PARTITIONING DEFECTIVE 3 HOMOLOG (C. ELEGANS)	1.52 ± 0.07	1.12 ± 0.04	1.05 ± 0
BM924824	RAS-RELATED C3 BOTULINUM TOXIN SUBSTRATE 1 (RHO FAMILY, SMALL GTP BINDING PROTEIN RAC1)	1.39 ± 0	1.41 ± 0.06	1.28 ± 0.18
W24540	FERM, RHOGEF AND PLECKSTRIN DOMAIN PROTEIN 2	1.28 ± 0.06	1.37 ± 0.1	1.46 ± 0.05
BI553762	CATENIN (CADHERIN-ASSOCIATED PROTEIN), ALPHA 2	1.36 ± 0.06	1.17 ± 0.03	1.88 ± 0.21
R35167	BAI1-ASSOCIATED PROTEIN 2	1.46 ± 0.15	0.97 ± 0.02	1.32 ± 0.12
T82805	PROTEIN TYROSINE PHOSPHATASE, RECEPTOR TYPE, J	1.49 ± 0.09	1.35 ± 0.13	1.19 ± 0.08
AA069424	INSULIN-LIKE GROWTH FACTOR 1 RECEPTOR	1.5 ± 0.09	1.16 ± 0.07	1.02 ± 0.05
N77112	IQ MOTIF CONTAINING GTPASE ACTIVATING PROTEIN 1	1.6 ± 0.35	1.54 ± 0.13	1.48 ± 0.04
BQ017489	ACTININ, ALPHA 1	1.57 ± 0.22	1.24 ± 0.1	1.5 ± 0.1
AA005247	MET PROTO-ONCOGENE (HEPATOCYTE GROWTH FACTOR RECEPTOR)	1.68 ± 0.34	1.3 ± 0.03	1.14 ± 0.07

For description see legend of table 5

**Table 8:** TGF-β regulated genes (36) in A549 involved in focal adhesion

**Acc No**	**DAVID Gene Name**	**1 hr**	**4 hr**	**12 hr**
N45505	VAV 1 ONCOGENE	0.64 ± 0.37	0.41 ± 0.11	0.31 ± 0.01
AA046597	INTEGRIN, BETA 4	1.26 ± 0.21	1.1 ± 0.19	1.8 ± 0.18
R52046	ACTININ, ALPHA 4	1.26 ± 0.68	1.21 ±	1.81 ± 0.21
AA030029	PROTEIN KINASE C, ALPHA	1.33 ± 0.23	1.56 ± 0.08	1.36 ± 0.03
BE738680	RAS HOMOLOG GENE FAMILY, MEMBER A	1.24 ± 0.11	1.4 ± 0.06	1.51 ± 0.03
H73472	CELL DIVISION CYCLE 42 (GTP BINDING PROTE IN, 25 KDA)	1.24 ± 0.27	1.33 ± 0.07	1.56 ± 0.01
W17002	INTEGRIN, ALPHA V (VITRONECTIN RECEPTOR, ALPHA POLYPEPTIDE, ANTIGEN CD51)	1.21 ± 0.06	2.32 ± 0.29	2.73 ± 0.28
AV747676	V-AKTMURINE THYMOMA VIRAL ONCOGENE HOMOLOG 3 (PROTEIN KINASE B, GAMMA)	1.06 ± 0.13	1.46 ± 0.05	1.18 ± 0.01
BM912016	P21(CDKN1A)-ACTIVATED KINASE 4	1.29 ± 0.03	1.1 ± 0.23	1.54 ± 0.03
BM008467	ZYXIN	0.95 ± 0.1	1.16 ± 0.14	1.41 ± 0.07
AA037738	HEPATOCYTE GROWTH FACTOR (HEPAPOIETIN A; SCATTER FACTOR)	1.43 ± 0.07	1.36 ± 0.03	1.06 ± 0.13
H89256	LAMININ, BETA 1	1.44 ± 0.02	1.4 ± 0.02	1.12 ± 0.07
AA026831	KINASE INSERT DOMAIN RECEPTOR (A TYPE III RECEPTOR TYROSINE KINASE)	1.17 ± 0.1	1.34 ± 0.02	1.06 ± 0.01
BM924824	RAS-RELATED C3 BOTULINUM TOXIN SUBSTRATE 1 (RHO FAMILY, SMALL GTP BINDING PROTEIN RAC1)	1.39 ± 0	1.41 ± 0.06	1.28 ± 0.18
AA122291	COLLAGEN, TYPE V, ALPHA 2	1.1 ± 0.08	1.19 ± 0.13	1.43 ± 0.11
R36401	FILAMIN B, BETA (ACTIN BINDING PROTEIN 278)	0.96 ± 0.02	1.17 ± 0.11	1.33 ± 0.01
R20452	CALPAIN 10	1.19 ± 0.21	1.08 ± 0.06	1.5 ± 0.01
H15287	P21(CDKN1A)-ACTIVATED KINASE 6	1.47 ± 0.08	1.21 ± 0.05	1.57 ± 0.04
W24540	FERM, RHOGEF AND PLECKSTRIN DOMAIN PROTEIN 2	1.28 ± 0.06	1.37 ± 0.1	1.46 ± 0.05
T39460	PROTEIN PHOSPHATASE 1, CATALYTIC SUBUNIT, ALPHA ISOFORM	1.35 ± 0.17	1.38 ± 0.04	1.49 ± 0.15
AA056664	V-AKT MURINE THYMOMA VTRAL ONCOGENE HOMOLOG 1	1.34 ± 0.15	1.44 ± 0.05	1.36 ± 0.07
AA127854	INTEGRIN, ALPHA 5 (FIBRONECTIN RECEPTOR, ALPHA POLYPEPTIDE)	1.4 ± 0.08	1.12 ± 0.03	1.22 ± 0.06
BG497332	THROMBOSPONDIN 1	0.87 ± 0.01	1.1 ± 0.1	2.24 ± 0.11
R25275	GLYCOGEN SYNTHASE KINASE 3 BETA.	1.15 ± 0.16	1.36 ± 0.04	1.23 ± 0.06
BQ016959	COLLAGEN, TYPE IV, ALPHA 2	1.18 ± 0.07	1.3 ± 0	1.33 ± 0.09
BM999610	V-JUN SARCOMA VIRUS 17 ONCOGENE HOMOLOG (AVIAN)	1.57 ± 0.26	2.13 ± 0.01	1.66 ± 0.18
AA044582	INTEGRIN, BETA 8	0.33 ± 0.03	0.26 ± 0.04	0.25 ± 0
T75436	MITOGEN-ACTIVATED PROTEIN KINASE 10	1.16 ± 0.17	1.22 ± 0.05	1.37 ± 0.07
AA069424	INSULIN-LIKE GROWTH FACTOR 1 RECEPTOR	1.5 ± 0.09	1.16 ± 0.07	1.02 ± 0.05
BI752921	PLATELET-DERIVED GROWTH FACTOR RECEPTOR, BETA POLYPEPTIDE	1.43 ± 0.09	1.03 ± 0.02	1.09 ± 0.07
N99223	INTEGRIN, ALPHA 9	1.46 ± 0.12	1.08 ±	1.29 ± 0.01
BQ017489	ACTININ, ALPHA 1	1.57 ± 0.22	1.24 ± 0.1	1.5 ± 0.1
AA010526	GLUCOCORTICOID RECEPTOR DNA BINDING FACTOR 1	1.44 ± 0.14	1.03 ± 0.04	1.31 ± 0
R14058	PROTEIN KINASE C, BETA 1	1.54 ± 0.06	1.08 ± 0.01	1.15 ± 0
AA005247	MET PROTO-ONCOGENE (HEPATOCYTE GROWTH FACTOR RECEPTOR)	1.68 ± 0.34	1.3 ± 0.03	1.14 ± 0.07
AA043909	PTK2 PROTEIN TYROSINE KINASE 2	0.52 ± 0.11	0.52 ± 0.12	0.34 ± 0

For description see legend of table 5

**Table 9:** TGF-β regulated genes (26) in A549 involved in insulin signalling

**Acc No**	**DAVID Gene Name**	**1 hr**	**4 hr**	**12 hr**
R23436	PROTEIN KINASE, CAMP-DEPENDENT, REGULATORY, TYPE II, ALPHA	0.32 ± 0.03	0.52 ± 0.03	0.22 ± 0.01
BI855956	LIPASE, HORMONE-SENSITIVE	1.17 ± 0.06	1.12 ± 0.1	1.38 ± 0.07
W32908	FORKHEAD BOX O1A (RHABDOMYOSARCOMA)	0.46 ± 0.17	0.38 ± 0.01	0.3 ± 0.05
R48236	SUPPRESSOR OF CYTOKINE SIGNALING 3	1.02 ± 0.21	1.51 ± 0	1.45 ± 0.09
BF034127	INSULIN RECEPTOR SUBSTRATE 2	0.3 ± 0.2	0.2 ± 0.09	0.33 ± 0.01
BG502582	CALMODULIN 1 (PHOSPHORYLASE KINASE, DELTA)	0.89 ± 0.02	1.41 ± 0.07	1.21 ± 0.14
R74161	PHOSPHORYLASE, GLYCOGEN; LIVER (HERS DISEASE, GLYCOGEN STORAGE DISEASE TYPE VI)	0.23 ± 0.06	0.28 ± 0.08	0.28 ± 0.02
AA030048	PROTEIN KINASE, CAMP-DEPENDENT, REGULATORY, TYPE I, BETA	1.1 ± 0.03	1.02 ± 0.06	1.31 ± 0.01
BM556894	RAPTOR	0.89 ±	1.34 ± 0.04	1.29 ± 0.19
AV747676	V-AKT MURINE THYMOMA VIRAL ONCOGENE HOMOLOG 3 (PROTEIN KINASE B, GAMMA)	1.06 ± 0.13	1.46 ± 0.05	1.18 ± 0.01
AA127934	PROTEIN KINASE, X-LINKED	1.21 ± 0.07	1.38 ± 0.01	1.35 ± 0.07
H89206	PROTEIN KINASE, CAMP-DEPENDENT, CATALYTIC, BETA	1.18 ± 0.08	1.42 ± 0.04	1.25 ± 0.03
W35243	PHOSPHOFRUCTOKINASE, PLATELET	1.15 ± 0.05	1.39 ± 0.06	1.34 ± 0.17
T93745	EUKARYOTIC TRANSLATION INITIATION FACTOR 4E MEMBER 2	1.4 ± 0	1.09 ± 0.11	1.06 ± 0.03
T39460	PROTEIN PHOSPHATASE 1, CATALYTIC SUBUNIT, ALPHA ISOFORM	1.35 ± 0.17	1.38 ± 0.04	1.49 ± 0.15
H80740	PHOSPHOFRUCTOKINASE, MUSCLE	1.42 ± 0.05	1.25 ± 0.04	1.36 ± 0.14
AA056664	V-AKT MURINE THYMOMA VIRAL ONCOGENE HOMOLOG 1	1.34 ± 0.15	1.44 ± 0.05	1.36 ± 0.07
H52478	TUBEROUS SCLEROSIS 2	1.23 ± 0.2	1.32 ± 0.05	1.4 ± 0.01
R25275	GLYCOGEN SYNTHASE KINASE 3 BETA	1.15 ± 0.16	1.36 ± 0.04	1.23 ± 0.06
H64260	PROTEIN KINASE, AMP-ACTIVATED, GAMMA 2 NON-CATALYTIC SUBUNIT	1.69 ±	1.21 ±	1.46 ± 0.05
T75436	MITOGEN-ACTIVATED PROTEIN KINASE 10	1.16 ± 0.17	1.22 ± 0.05	1.37 ± 0.07
T75267	FK506 BINDING PROTEIN 12-RAPAMYCIN ASSOCIATED PROTEIN 1	1.77 ± 0.24	1.37 ± 0.05	1.2 ± 0.06
H44888	SUPPRESSOR OF CYTOKINE SIGNALING 6	0.25 ± 0.13	0.16 ±	0.18 ± 0.01
H59587	ACETYL-COENZYME A CARBOXYLASE ALPHA	1.42 ± 0.44	1.32 ± 0.01	1.18 ± 0
H08429	TUBEROUS SCLEROSIS 1	1.43 ± 0.09	1.09 ± 0.07	1.35 ± 0.1
AA010882	GLYCOGEN SYNTHASE 1 (MUSCLE)	1.46 ± 0.07	1.15 ± 0	1.01 ± 0.08

For description see legend of table 5

**Table 10:** TGF-β regulated genes (36) in A549 involved in regulation of actin cytoskeleton

**Acc No**	**DAVID Gene Name**	**1 hr**	**4 hr**	**12 hr**
H83405	FYVE, RHOGEF AND PH DOMAIN CONTAINING 1 (FACIOGENITAL DYSPLASIA)	0.57 ± 0.41	0.35 ±	0.26 ± 0.01
N45505	VAV 1 ONCOGENE	0.64 ± 0.37	0.41 ± 0.11	0.31 ± 0.01
N45518	NCK-ASSOCIATED PROTEIN 1	1.03 ± 0.12	1.76 ± 0.2	1.39 ± 0.18
AA046597	INTEGRIN, BETA 4	1.26 ± 0.21	1.1 ± 0.19	1.8 ± 0.18
R52046	ACTININ, ALPHA 4	1.26 ± 0.68	1.21 ±	1.81 ± 0.21
N42722	GUANINE NUCLEOTIDE BINDING PROTEIN (G PROTEIN), GAMMA 12	1.44 ± 0.04	1.5 ± 0.09	1.28 ± 0.17
AL598940	MYOSIN, LIGHT POLYPEPTIDE 1, ALKALI; SKELETAL, FAST	1.15 ± 0.01	1.33 ± 0.17	1.56 ± 0.02
BE738680	RAS HOMOLOG GENE FAMILY, MEMBER A	1.24 ± 0.11	1.4 ± 0.06	1.51 ± 0.03
H73472	CELL DIVISION CYCLE 42 (GTP BINDING PROTEIN, 25KDA)	1.24 ± 0.27	1.33 ± 0.07	1.56 ± 0.01
W17002	INTEGRIN, ALPHA V (VITRONECTIN RECEPTOR, ALPHA POLYPEPTIDE, ANTIGEN CD51)	1.21 ± 0.06	2.32 ± 0.29	2.73 ± 0.28
AA088648	FIBROBLAST GROWTH FACTOR RECEPTOR 1 (FMS-RELATED TYROSINE KINASE 2, PFEIFFER SYNDROME)	1.45 ± 0.24	1.97 ± 0.08	1.77 ± 0.09
N57424	T-CELL LYMPHOMA INVASION AND METASTASIS 1	1.35 ± 0.02	1.23 ± 0.04	1.34 ± 0.02
H10616	PROFILIN 2	1.44 ± 0.08	1.49 ± 0.11	1.1 ± 0
BM912016	P21(CDKN1A)-ACTIVATED KINASE 4	1.29 ± 0.03	1.1 ± 0.23	1.54 ± 0.03
H23109	FIBROBLAST GROWTH FACTOR 1 (ACIDIC)	0.94 ±	1.36 ± 0.01	1.13 ± 0.05
BM924824	RAS-RELATED C3 BOTULINUM TOXIN SUBSTRATE 1 (RHO FAMILY, SMALL GTP BINDING PROTEIN RAC1)	1.39 ± 0	1.41 ± 0.06	1.28 ± 0.18
H15287	P21(CDKN1A)-ACTIVATED KINASE 6	1.47 ± 0.08	1.21 ± 0.05	1.57 ± 0.04
T39460	PROTEIN PHOSPHATASE 1, CATALYTIC SUBUNIT, ALPHA ISOFORM	1.35 ± 0.17	1.38 ± 0.04	1.49 ± 0.15
BM888157	THYMOSIN, BETA 4, X-LINKED	1.02 ±	1.58 ±	1.93 ± 0.14
T83174	ACTIN RELATED PROTEIN 2/3 COMPLEX, SUBUNIT 1B, 41KDA	1.24 ± 0.08	1.06 ± 0.1	1.54 ± 0.04
AA127854	INTEGRIN, ALPHA 5 (FIBRONECTIN RECEPTOR, ALPHA POLYPEPTIDE)	1.4 ± 0.08	1.12 ± 0.03	1.22 ± 0.06
H27657	G PROTEIN-COUPLED RECEPTOR KINASE INTERACTOR 1	1.39 ± 0.09	1.11 ± 0.04	1.34 ± 0.25
N29131	MOESIN	1.37 ± 0.09	1.03 ± 0.06	1.42 ± 0.05
AA044582	INTEGRIN, BETA 8	0.33 ± 0.03	0.26 ± 0.04	0.25 ± 0
T77476	GUANINE NUCLEOTIDE BINDING PROTEIN (G PROTEIN), ALPHA 13	1.39 ± 0.41	1.59 ± 0.08	1.24 ± 0.14
R35167	BAI1-ASSOCIATED PROTEIN 2	1.46 ± 0.15	0.97 ± 0.02	1.32 ± 0.12
H06909	PROTEIN PHOSPHATASE 1, REGULATORY (INHIBITOR) SUBUNIT 12B	1.34 ± 0.03	1.16 ± 0.01	1.08 ± 0.03
H14999	RAC/CDC42 GUANINE NUCLEOTIDE EXCHANGE FACTOR (GEF) 6	0.22 ± 0.13	0.27 ± 0.14	0.28 ± 0
N77112	IQ MOTIF CONTAINING GTPASE ACTIVATING PROTEIN 1	1.6 ± 0.35	1.54 ± 0.13	1.48 ± 0.04
BI752921	PLATELET-DERIVED GROWTH FACTOR RECEPTOR, BETA POLYPEPTIDE	1.43 ± 0.09	1.03 ± 0.02	1.09 ± 0.07
N99223	INTEGRIN, ALPHA 9	1.46 ± 0.12	1.08 ±	1.29 ± 0.01
BI258438	COFILIN 1 (NON-MUSCLE)	1.24 ± 0.09	1.08 ± 0.09	1.42 ± 0.04
BQ017489	ACTININ, ALPHA 1	1.57 ± 0.22	1.24 ± 0.1	1.5 ± 0.1
H51445	LIM DOMAIN KINASE 1	1.3 ± 0	1.02 ± 0.11	1.12 ± 0.01
AA010526	GLUCOCORTICOID RECEPTOR DNA BINDING FACTOR 1	1.44 ± 0.14	1.03 ± 0.04	1.31 ± 0
AA043909	PTK2 PROTEIN TYROSINE KINASE 2	0.52 ± 0.11	0.52 ± 0.12	0.34 ± 0

For description see legend of table 5

**Table 11:** TGF-β regulated genes (23) in A549 involved in Wnt signalling

**Acc No**	**DAVID Gene Name**	**1 hr**	**4 hr**	**12 hr**
H78804	SMAD, MOTHERS AGAINST DPP HOMOLOG 3 (DROSOPHILA)	1.22 ± 0.09	1.36 ± 0.02	1.15 ± 0.06
AA029506	CALCIUM/CALMODULIN-DEPENDENT PROTEIN KINASE (CAM KINASE) II DELTA	1.29 ± 0.22	1.41 ± 0.07	1.44 ± 0.02
AA027986	PROTEIN PHOSPHATASE 2 (FORMERLY 2A), CATALYTIC SUBUNIT, ALPHA ISOFORM	1.24 ± 0.15	1.65 ± 0.03	1.34 ± 0.11
AA029516	TRANSCRIPTION FACTOR 7-LIKE 2 (T-CELL SPECIFIC, HMG-BOX)	1.25 ± 0.02	1.41 ± 0.02	1.14 ± 0.2
AA044665	F-BOX AND WD-40 DOMAIN PROTEIN 11	1.09 ± 0.02	1.31 ± 0.02	1.07 ± 0.1
AA030029	PROTEIN KINASE C, ALPHA	1.33 ± 0.23	1.56 ± 0.08	1.36 ± 0.03
BE738680	RAS HOMOLOG GENE FAMILY, MEMBER A	1.24 ± 0.11	1.4 ± 0.06	1.51 ± 0.03
R55134	TRANSCRIPTION FACTOR 7-LIKE 1 (T-CELL SPECIFIC, HMG-BOX)	1.45 ± 0.03	1.2 ± 0.1	1.27 ± 0.05
W49562	PHOSPHOLIPASE C, BETA 4	1.26 ± 0.25	1.27 ± 0.04	1.77 ± 0.27
AA127934	PROTEIN KINASE, X-LINKED	1.21 ± 0.07	1.38 ± 0.01	1.35 ± 0.07
H89206	PROTEIN KINASE, CAMP-DEPENDENT, CATALYTIC, BETA	1.18 ± 0.08	1.42 ± 0.04	1.25 ± 0.03
N72642	CALCYCLIN BINDING PROTEIN	1.38 ± 0.01	1.5 ± 0.12	1.08 ± 0.06
BM924824	RAS-RELATED C3 BOTULINUM TOXIN SUBSTRATE 1 (RHO FAMILY, SMALL GTP BINDING PROTEIN RAC1)	1.39 ± 0	1.41 ± 0.06	1.28 ± 0.18
W51811	WINGLESS-TYPE MMTV INTEGRATION SITE FAMILY, MEMBER 5A	1.48 ± 0.1	1.54 ± 0.06	1.51 ± 0.19
BM311217	WINGLESS-TYPE MMTV INTEGRATION SITE FAMILY, MEMBER 4	1.63 ±	0.97 ±	1.5 ± 0.02
R25275	GLYCOGEN SYNTHASE KINASE 3 BETA	1.15 ± 0.16	1.36 ± 0.04	1.23 ± 0.06
AA152025	FRIZZLED HOMOLOG 4 (DROSOPHILA)	1.47 ± 0.28	1.56 ± 0.14	1.27 ± 0.12
BM999610	V-JUN SARCOMA VIRUS 17 ONCOGENE HOMOLOG (AVIAN)	1.57 ± 0.26	2.13 ± 0.01	1.66 ± 0.18
T75436	MITOGEN-ACTIVATED PROTEIN KINASE 10	1.16 ± 0.17	1.22 ± 0.05	1.37 ± 0.07
AA028175	CULLIN 1	1.51 ± 0.15	1.15 ± 0.04	0.96 ± 0.02
H70045	CALCIUM/CALMODULIN-DEPENDENT PROTEIN KINASE (CAM KINASE) II GAMMA	1.45 ± 0.08	1.39 ± 0.16	1.21 ± 0.12
R72188	LOW DENSITY LIPOPROTEIN RECEPTOR-RELATED PROTEIN 5	1.27 ± 0.13	0.87 ±	1.38 ± 0.02
R14058	PROTEIN KINASE C, BETA 1	1.54 ± 0.06	1.08 ± 0.01	1.15 ± 0

For description see legend of table 5
