# Supplementary figures and images for: Expression profiling of genes regulated by TGF-beta: Differential regulation in normal and tumour cells
Source: BMC Genomics. 2007 Apr 11;8:98. doi: 10.1186/1471-2164-8-98 (PMC1858692; doi:10.1186/1471-2164-8-98)

## Slide 1
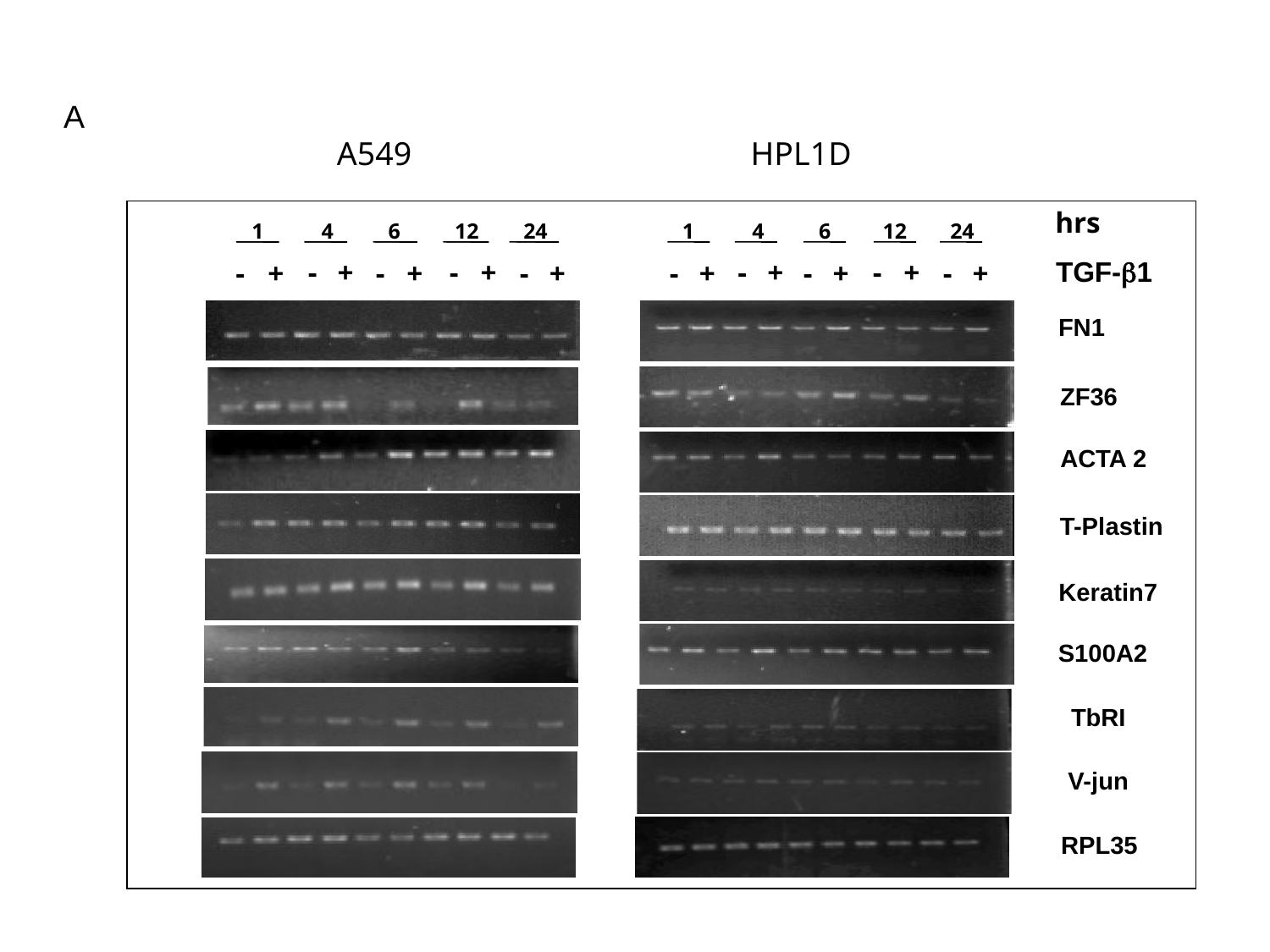

A
A549
HPL1D
hrs
1
4
12
6
24
-
+
-
+
-
+
-
+
-
+
1
4
12
6
24
-
+
-
+
-
+
-
+
-
+
TGF-β1
FN1
ZF36
ACTA 2
T-Plastin
Keratin7
S100A2
TbRI
V-jun
RPL35

## Slide 2
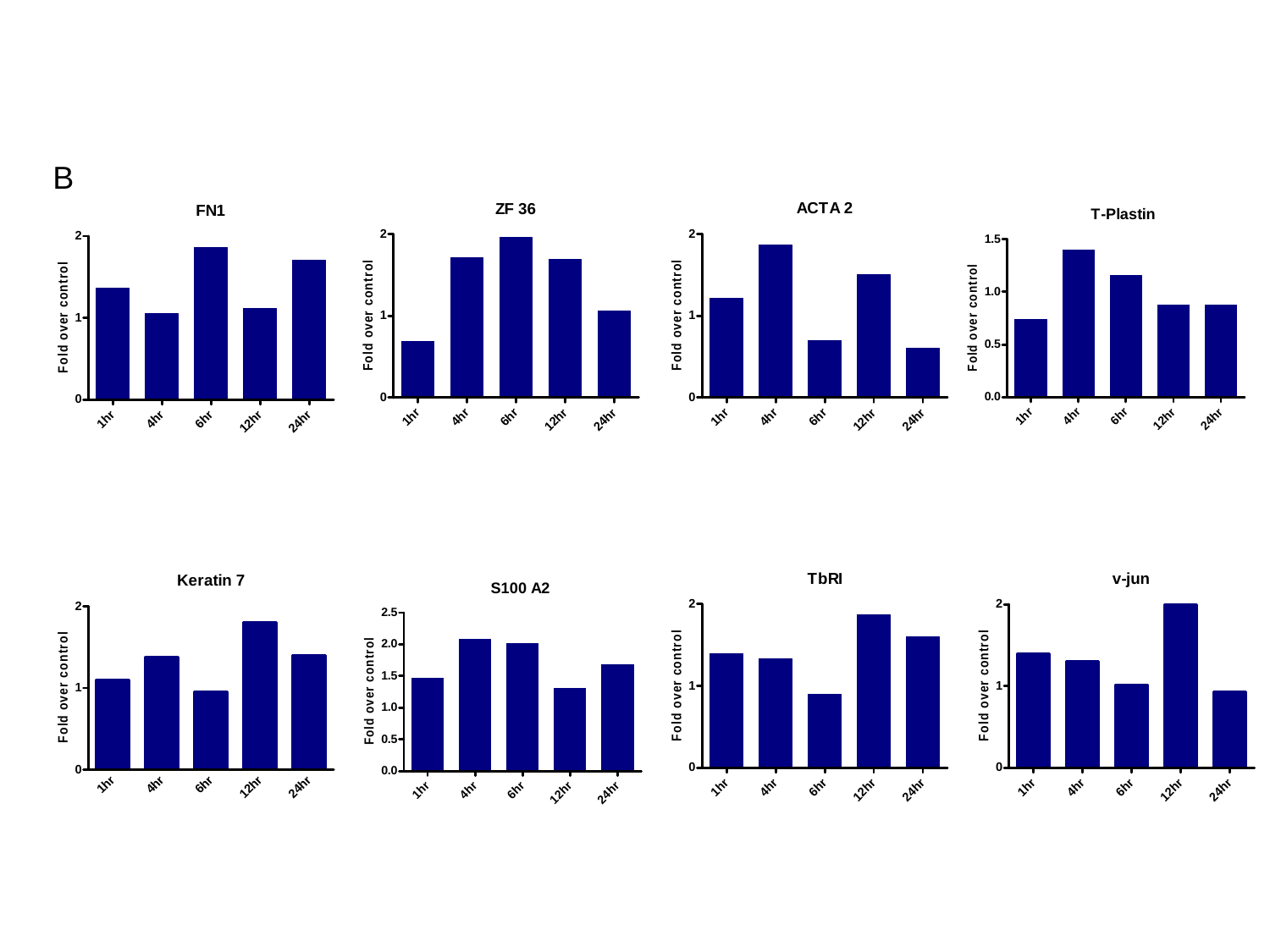

B

## Slide 3
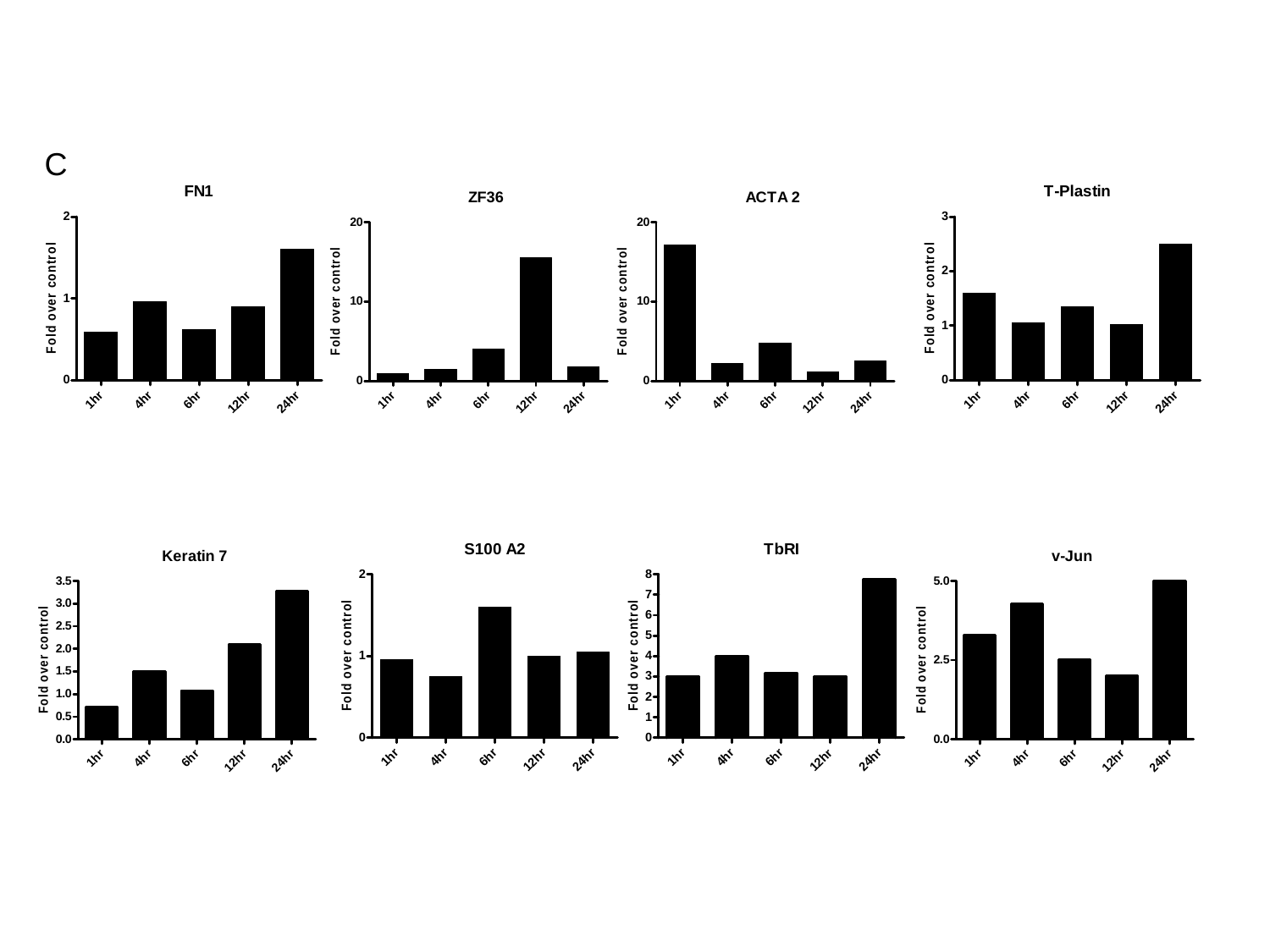

C

Supplement: Additional file 4 — Semi-quantitative RT-PCR analyses of selected genes with respect to regulation by TGF-β in A549 and HPL1D cells. The respective cell-lines were grown to 90% confluence (as described in methods section), washed with serum free medium, treated with 5 ng/ml TGF-β 1 for 1, 4, 6, 12 and 24 hours. Each treatment also has untreated cells as control at each time-point. Two microgram of total RNA from each treatment was reverse transcribed and cDNA equivalent to 20 ng total RNA was used for the PCR reactions. All PCR reactions were done under non saturating conditions. The products were resolved on 2% agarose gel and the gel pictures were taken on Kodak Image station 440CF. The band intensities were quantified using Kodak 1D 3.6 software. A, ethidium bromide staining pattern of the PCR products. B and C graphs representing the fold change over untreated controls after normalization with the expression of RPL35a, in HPL1D and A549 cells respectively. [file 1471-2164-8-98-S4.ppt]

## Slide 1
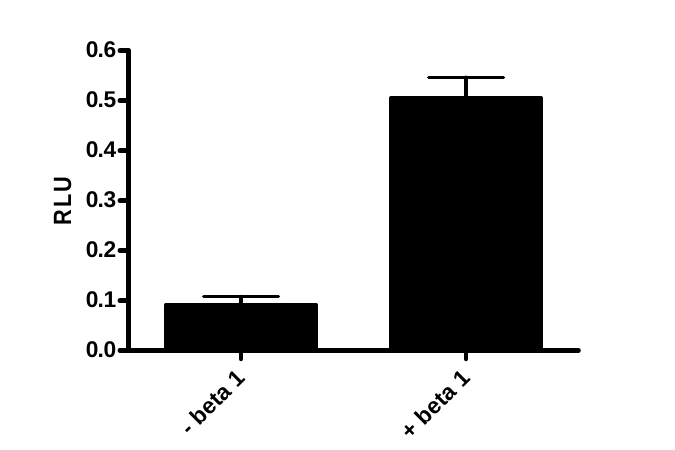

Supplement: Additional file 5 — Induction of pSBE-Luc activity by TGF-β in A549 cells. For SBE-luc induction by TGF-β in A549 cells, twenty five thousand cells were plated in 24 well dishes 16–24 hours prior to transfection. 200 ng of pSBE-luc plasmid and 1.25 ng of pRL-CMV construct (Renilla luciferase, for transfection normalization) were transfected using Effectene reagent (Qiagen GmbH, Germany) in serum free conditions for 12 hrs. The cells were recovered in medium containing 10% FBS for 24 hours, washed with serum free medium for 24 hours (3 changes) and then treated with 5 ng/ml TGF-β for 18 hours. The cells were then lysed and lysates were used for dual-luciferase assay (Promega inc, USA). The ratio of the firefly-luciferase to renilla-luciferase has been plotted on the y-axis. [file 1471-2164-8-98-S5.ppt]
